# Use of Machine Learning for Predicting Escitalopram Treatment Outcome From Electroencephalography Recordings in Adult Patients With Depression

**DOI:** 10.1001/jamanetworkopen.2019.18377

**Published:** 2020-01-03

**Authors:** Andrey Zhdanov, Sravya Atluri, Willy Wong, Yasaman Vaghei, Zafiris J. Daskalakis, Daniel M. Blumberger, Benicio N. Frey, Peter Giacobbe, Raymond W. Lam, Roumen Milev, Daniel J. Mueller, Gustavo Turecki, Sagar V. Parikh, Susan Rotzinger, Claudio N. Soares, Colleen A. Brenner, Fidel Vila-Rodriguez, Mary Pat McAndrews, Killian Kleffner, Esther Alonso-Prieto, Stephen R. Arnott, Jane A. Foster, Stephen C. Strother, Rudolf Uher, Sidney H. Kennedy, Faranak Farzan

**Affiliations:** 1 School of Mechatronic Systems Engineering, Simon Fraser University, Surrey, British Columbia, Canada; 2 Centre for Engineering-Led Brain Research, Simon Fraser University, Surrey, British Columbia, Canada; 3 Temerty Centre for Therapeutic Brain Intervention, Centre for Addiction and Mental Health, Toronto, Ontario, Canada; 4 Institute of Biomaterial and Biomedical Engineering, Toronto, Ontario, Canada; 5 The Edward S. Rogers Sr Department of Electrical & Computer Engineering, University of Toronto, Toronto, Ontario, Canada; 6 Department of Psychiatry, University of Toronto, Toronto, Ontario, Canada; 7 Institute of Medical Science, Faculty of Medicine, University of Toronto, Toronto, Ontario, Canada; 8 Department of Psychiatry and Behavioural Neurosciences, McMaster University, Hamilton, Ontario, Canada; 9 Mood Disorders Program and Women’s Health Concerns Clinic, St Joseph’s Healthcare Hamilton, Hamilton, Ontario, Canada; 10 Department of Psychiatry, University Health Network, University of Toronto, Toronto, Ontario, Canada; 11 Department of Psychiatry, University of British Columbia, Vancouver, British Columbia, Canada; 12 Departments of Psychiatry and Psychology, Queen’s University, Providence Care Hospital, Kingston, Ontario, Canada; 13 Department of Psychiatry, McGill University, Montreal, Quebec, Canada; 14 Department of Psychiatry, University of Michigan, Ann Arbor; 15 Li Ka Shing Knowledge Institute, St Michael's Hospital, Toronto, Ontario, Canada; 16 Department of Psychiatry, Queen’s University, Kingston, Ontario, Canada; 17 Department of Psychology, Loma Linda University, Loma Linda, California; 18 Krembil Research Institute, University Health Network, Toronto, Ontario, Canada; 19 Rotman Research Institute, Baycrest Centre, Toronto, Ontario, Canada; 20 St Michael’s Hospital, Toronto, Ontario, Canada; 21 Department of Medical Biophysics, University of Toronto, Toronto, Ontario, Canada; 22 Department of Psychiatry, Dalhousie University, Halifax, Nova Scotia, Canada; 23 Institute of Psychiatry, Psychology & Neuroscience, Social, Genetic and Developmental Psychiatry Centre, King’s College London, London, United Kingdom

## Abstract

**Importance:**

Social and economic costs of depression are exacerbated by prolonged periods spent identifying treatments that would be effective for a particular patient. Thus, a tool that reliably predicts an individual patient’s response to treatment could significantly reduce the burden of depression.

**Objective:**

To estimate how accurately an outcome of escitalopram treatment can be predicted from electroencephalographic (EEG) data on patients with depression.

**Design, Setting, and Participants:**

This prognostic study used a support vector machine classifier to predict treatment outcome using data from the first Canadian Biomarker Integration Network in Depression (CAN-BIND-1) study. The CAN-BIND-1 study comprised 180 patients (aged 18-60 years) diagnosed with major depressive disorder who had completed 8 weeks of treatment. Of this group, 122 patients had EEG data recorded before the treatment; 115 also had EEG data recorded after the first 2 weeks of treatment.

**Interventions:**

All participants completed 8 weeks of open-label escitalopram (10-20 mg) treatment.

**Main Outcomes and Measures:**

The ability of EEG data to predict treatment outcome, measured as accuracy, specificity, and sensitivity of the classifier at baseline and after the first 2 weeks of treatment. The treatment outcome was defined in terms of change in symptom severity, measured by the Montgomery-Åsberg Depression Rating Scale, before and after 8 weeks of treatment. A patient was designated as a responder if the Montgomery-Åsberg Depression Rating Scale score decreased by at least 50% during the 8 weeks and as a nonresponder if the score decrease was less than 50%.

**Results:**

Of the 122 participants who completed a baseline EEG recording (mean [SD] age, 36.3 [12.7] years; 76 [62.3%] female), the classifier was able to identify responders with an estimated accuracy of 79.2% (sensitivity, 67.3%; specificity, 91.0%) when using only the baseline EEG data. For a subset of 115 participants who had additional EEG data recorded after the first 2 weeks of treatment, use of these data increased the accuracy to 82.4% (sensitivity, 79.2%; specificity, 85.5%).

**Conclusions and Relevance:**

These findings demonstrate the potential utility of EEG as a treatment planning tool for escitalopram therapy. Further development of the classification tools presented in this study holds the promise of expediting the search for optimal treatment for each patient.

## Key Points

**Question:**

Is it possible to predict whether the condition of a patient with depression will improve after escitalopram treatment by analyzing their resting-state electroencephalographic signals?

**Findings:**

In this prognostic study of data from 122 patients diagnosed with major depressive disorder, support vector machine classifiers demonstrated an accuracy of 82.4% for predicting escitalopram treatment outcome.

**Meaning:**

When complemented by appropriate analysis methods, resting-state electroencephalographic recordings may be instrumental in improving treatment of patients with depression.

## Teaser

This prognostic study of patients with major depressive disorder estimates how accurately an outcome of escitalopram treatment can be predicted from electroencephalographic data.

## Introduction

Antidepressant medications are the first-line treatments for patients with major depressive disorder (MDD). However, remission rates are approximately 30% to 40% after 1 medication trial and approximately 50% to 55% after a second separate trial^1^ and decline progressively with subsequent medication trials.^2^ Because of the heterogeneity of depression and the lack of consensus on the precise mechanism of action of antidepressants, matching patients to effective treatments has been a daunting task for practitioners. Currently, practitioners use a prolonged trial-and-error process to identify the optimal antidepressant for each patient, with patients often spending months to years experiencing distressing symptoms.^2,3^ Although clinical interviews and scales are available to confirm the diagnosis and severity of symptoms, they are not sufficient for selecting an appropriate treatment for each patient.^4,5^ One solution that might help to reduce the time spent in failed trials and accompanying personal and economic burden is to identify biological predictors of response to an antidepressant. A personalized tool for the prediction of response to antidepressants may expedite the treatment and lead to faster relief of symptoms.

One promising technique for identifying biological predictors of response to antidepressant treatment is electroencephalography (EEG), which records the oscillations of brain electric potentials measured from electrodes attached to the scalp. These potentials are produced by the synchronized activity of large (thousands to millions of neurons) neuronal populations inside the brain.^6^ Converging lines of evidence suggest that features derived from EEG recordings before treatment may predict subsequent clinical response to antidepressants.^7,8,9,10,11^ Several EEG studies^12,13,14,15,16,17,18^ have reported that characteristics of resting-state neural oscillations, especially in the alpha and theta bands, may be used to predict the response to antidepressants. Power of posterior alpha activity has been associated with response to fluoxetine and amitriptyline^19,20^; theta activity with response to imipramine, venlafaxine, and several selective serotonin reuptake inhibitors^18,21,22^; delta activity with response to imipramine and paroxetine^18,23^; interhemispheric delta asymmetry with response to fluoxetine^24^; and increased delta activity in the rostral anterior cingulate cortex with response to nortriptyline, fluoxetine, and venlafaxine.^25,26^ Several studies^27,28,29^ have also evaluated the association between nonlinear features of EEG signals (eg, complexity or variability of neural dynamics) and response to antidepressants, such as citalopram, clomipramine, escitalopram, bupropion, and mirtazapine.

The aforementioned works provide compelling evidence that resting-state EEG can be used to predict response to antidepressant medication. However, they fail to address several questions that are important for translating this discovery into a clinical tool. First, they restrict themselves to relatively small and homogeneous feature sets; that is, they do not directly compare large (several thousands and more) numbers of features that represent different perspectives on the EEG signals (eg, spectral power density, entropy, and microstates). Second, many studies report poor prediction accuracy. Third, the estimates of the prediction accuracy reported by the studies are typically biased upward by the lack of an independent testing set. Many of the studies fail to control this bias by appropriate techniques, such as cross-validation.

The shortcomings of the previous studies^7,15,16,17,19^ stem, to a considerable degree, from relatively small sample sizes available to their authors, typically ranging from 12 to 50 participants. The research described in this publication uses data from a large, Canada-wide, multicenter study, the first Canadian Biomarker Integration Network in Depression (CAN-BIND-1) study,^30^ to overcome this limitation and address some of the questions remaining from the previous research. In particular, the present study attempts to compare the predictive power of a wide range of diverse features and maximally reduce the bias of the estimate of the prediction accuracy by using cross-validation.

The current study evaluated the predictive power of resting-state EEG signals recorded at baseline (before the treatment was initiated) and 2 weeks after the start of the escitalopram treatment. From the clinical perspective, predicting the treatment outcome from the baseline EEG data alone is preferable because this approach would expedite treatment planning and eliminate the need for the second EEG examination. However, we hypothesized that inclusion of the second time point, 2 weeks after treatment initiation, would identify early changes in brain function that may be predictive of treatment outcome and therefore would result in increased prediction accuracy, potentially outweighing the costs of additional EEG examination. We also conjectured that use of the most predictive EEG features selected from a wide variety of possible candidates—both linear and nonlinear—would increase the predictive power of the model.

## Methods

In this prognostic study, we developed and validated a model that predicted treatment outcome from multivariate EEG data following the recommendations for the Transparent Reporting of a Multivariable Prediction Model for Individual Prognosis or Diagnosis (TRIPOD) reporting guideline.^31^ All the participants of the study provided written informed consent according to the ethics approval issued by the ethics review board at each of the participating sites.

### Data Acquisition and Preprocessing

#### Participants

We used EEG data from the CAN-BIND-1 study.^30^ All participants were between 18 and 60 years of age and met the *Diagnostic and Statistical Manual of Mental Disorders* (Fourth Edition) requirements for MDD and a current major depressive episode. The researchers that performed the machine learning analysis received only deidentified data. Clinical practitioners who conducted behavioral patient evaluations had access to patients’ personal details.

We analyzed resting-state recordings from 122 patients with MDD at baseline (before the treatment started) and 115 of the same patients 2 weeks after escitalopram treatment initiation. A patient was categorized as a responder if the Montgomery-Åsberg Depression Rating Scale (MADRS) score decreased by least 50% in the first 8 weeks of the treatment and as a nonresponder if the score decrease was less than 50% (more details are given in the eMethods in the Supplement).

#### Intersite Data Harmonization and Preprocessing

Because CAN-BIND-1 data were recorded at multiple sites, they were harmonized (converted into a common site-independent format) and then preprocessed to remove some common sources of noise, such as 60-Hz power line interference. Data harmonization and preprocessing are described in more detail in the eMethods in the Supplement.

### Feature Computation and Ranking

#### Feature Sources

Throughout the study, we compared 4 different sources of features that can be used to predict treatment outcome. The sources differed in the timing of the features used for the prediction: (1) baseline source comprised features derived from the data collected at baseline, (2) week 2 source included features derived from data collected 2 weeks after the start of treatment, (3) early change source denoted change in features from baseline to week 2, and (4) combined source combined features from baseline and early change.

#### Feature Definitions

We evaluated a diverse variety of potentially informative EEG features. In addition to traditional electrode-level frequency analysis, we considered power spectral features in the source domain,^26,32^ spatiotemporal complexity,^33,34^ and global brain network dynamics^35^ previously found to have predictive value for antidepressant response. The power spectral features used the following definitions of the frequency bands: delta (1-3.5 Hz), theta (4-8 Hz), low alpha (8.5-10 Hz), high alpha (10.5-12 Hz), low beta (12.5-18 Hz), middle beta (18.5-21 Hz), and high beta (21.5-30 Hz).

Four classes of features were used in the study. The first class was electrode-level spectral features (power within a given frequency band at each electrode) and lateralization of power for symmetrically located pairs of electrodes. The second class was source-level spectral features, which are estimates of power within a given frequency band for several locations within the brain obtained using the eLORETA source reconstruction algorithm.^36^ The third class was multiscale-entropy-based features, which are entropy estimates for individual electrodes as well as entropy asymmetry indexes for symmetrically located electrode pairs computed over 70 different timescales using a temporal coarse-graining process.^33,34^ The fourth class was microstate-based features, which are features derived from decomposition of EEG signals into microstates using established microstate segmentation procedures.^35,37^ More details on the feature definitions are given in the eMethods in the Supplement.

#### Feature Ranking

The feature definitions described result in a large feature set that contains thousands of variables. To construct a practical predictor of the treatment outcome, feature selection was performed to identify a small subset of the most informative features to be used with the final classifier.

Identifying the optimal feature set for machine learning is, in general, an unsolved problem.^38,39^ Machine learning literature suggests a number of different approaches, such as wrapper or filter methods, each with its own advantages and drawbacks. In this study, we adopted a filter-based approach to remove uninformative features, among other reasons, because filter methods are less prone to overfitting and easy to compute.^40^

We used a filtering method based on an unpaired 2-tailed *t* test (described in detail in the eMethods in the Supplement) to assign each feature a score of 0 to 100, with more votes indicating the features that are more robustly predictive of the treatment outcome. Feature selection involved selecting only the features that had a number of votes above the specified vote threshold *T*.

### Classifier Construction and Performance Estimation

After a small subset of features has been selected by applying a given threshold to the features’ rankings, a classifier from the selected features can be constructed. For the current study, we used radial basis function (RBF) kernel-based support vector machines (SVMs) for classification. This choice was motivated by the SVMs’ maturity and popularity in machine learning research in general and the success of the machines in the field of neuropsychiatry in particular.^41^

For each of the 4 feature sources (baseline, week 2, early change, and combined) and several different vote thresholds *T* ∈ {50, 60, 70, 80, 90}, we trained an RBF SVM classifier to achieve maximum balanced accuracy (mean of sensitivity and specificity) and estimated its performance using cross-validation. Classifier construction and performance estimation are described in more detail the eMethods in the Supplement.

### Assessment of Generalizability Across Sites

One important property of a machine learning model for predicting treatment outcome is the ability to generalize to the data obtained from a clinical site that was not involved in the training of the model. We assessed our approach’s capacity for such generalization by using the leave-one-site-out cross-validation procedure adapted from the literature.^42^ For each of the 4 participating sites, we performed the following computations. First, we partitioned all the data into the testing set that contained data from the given site and the training set that contained data from the 3 remaining sites. Second, we performed the same procedure as previously described (computing feature rankings, selecting the features with rankings above a certain threshold, and constructing the resulting RBF SVM classifier) using the training set only. We used *T* = 60 for the vote threshold because this value seemed to provide a good balance between the model’s complexity and performance. Third, we assessed the classifier’s performance using the testing set. This procedure yielded 4 different accuracy estimates (1 per each site).

### Statistical Analysis

Our study supplants classic statistical analysis by estimating the ability of SVMs to predict treatment outcome from the EEG data. The SVMs’ ability to differentiate between responders and nonresponders from the EEG data (estimated by cross-validation as described above) served as a statistical measure of difference between the 2 populations. The analysis was performed in MATLAB software (MathWorks) using the LIBSVM toolbox.^43^

## Results

### Participants

In the current study, we analyzed EEG data recorded before initiation of treatment from 122 participants (mean [SD] age, 36.3 [12.7] years; 76 [62.3%] female) (Table 1). The mean (SD) MADRS score for the 122 participants whose data were included in the analysis was 30.1 (5.8). In addition, for a subset of 115 of the 122 participants (mean [SD] age, 36.2 [12.4] years; 72 [62.6%] female), we analyzed EEG data recorded 2 weeks after the treatment started.

**Table 1.** Demographic and Clinical Data for Study Participants

Clinical or Demographic Variable	EEG Recording Site				All (N = 122)	All Responders at Week 8 (n = 55)	All Nonresponders at Week 8 (n = 67)
	UBC (n = 52)	TGH (n = 45)	QNS (n = 18)	CAM (n = 7)			
Age, mean (SD), y	35.4 (11.5)	35.7 (12.6)	42.7 (14.0)	30.4 (12.1)	36.3 (12.7)	36.0 (12.7)	36.6 (12.6)
Sex, No.							
Male	19	18	9	0	46	19	27
Female	33	27	9	7	76	36	40
MADRS score, mean (SD)							
Baseline	28.5 (5.8)	32.3 (5.6)	30.0 (4.6)	28.0 (4.8)	30.1 (5.8)	29.5 (5.8)	30.5 (5.8)
Week 2	21.9 (7.3)	25.1 (10.1)	23.1 (5.2)	21.1 (3.7)	23.2 (8.5)	20.1 (8.4)	25.8 (7.8)
Week 8	14.6 (9.2)	19.1 (12.0)	18.1 (9.9)	15.7 (5.6)	16.8 (10.5)	7.9 (5.0)	24.2 (7.7)
Decrease in MADRS score, mean (SD)							
Baseline to week 8	13.9 (9.2)	13.2 (11.8)	11.9 (9.9)	12.3 (4.1)	13.2 (10.2)	21.7 (6.7)	6.3 (6.8)
Baseline to week 8 compared with baseline, %	48.7 (32.7)	41.1 (3.8)	39.2 (34.9)	44.5 (15.7)	44.3 (32.6)	73.3 (16.0)	20.4 (21.6)
Responders as assessed at week 8, No. (%)	24 (46.2)	21 (46.7)	7 (38.9)	3 (42.9)	55 (45.1)	NA	NA
Nonresponders as assessed at week 8, No. (%)	28 (53.8)	24 (53.3)	11 (61.1)	4 (57.1)	67 (54.9)	NA	NA

Abbreviations: CAM, Centre for Addiction and Mental Health; EEG, electroencephalography; MADRS, Montgomery-Åsberg Depression Rating Scale; NA, not applicable; QNS, Queens University; TGH, Toronto General Hospital; UBC, University of British Columbia.

### Feature Computation and Ranking

**Figure 1.**
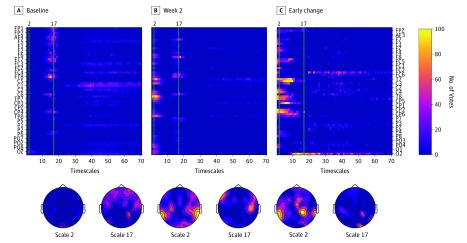
Number of Votes for Multiscale Entropy Features The figure shows rankings for only 3 of 4 feature sources: baseline (A), week 2 (B), and early change (C). Combined feature source was the union of baseline and early change sources; therefore, computing the rankings for the combined features yielded results that were similar to the results for the baseline and early change sources. For that reason, feature rankings for combined feature source are not shown. The matrices in the top row display rankings for all the features; the topographic plots (left ear is left, nose is up) in the bottom row show distribution over the scalp of the rankings for the features corresponding to multiscale entropy scales 2 and 17 (marked by vertical green lines in the top row plots).

Of all the different feature classes considered in this study, the most clearly structured pattern of informative features was exhibited by multiscale entropy. Figure 1 reveals several prominent elements of this pattern. There was a tight cluster of predictive features occurring around timescale 17. The features exhibited frontoparietal distribution over the electrode map and appeared mostly unchanged between the baseline and week 2 measurements, resulting in absence of any predictive early change features at this scale. Another prominent pattern was the cluster of predictive features at lower timescales (eg, around timescale 2). This more posteriorly distributed pattern was absent in the baseline measurement but emerged in the week 2 recording. This difference between baseline and week 2 was also reflected in the prominent cluster of predictive early change features appearing at lower timescales.

**Figure 2.**
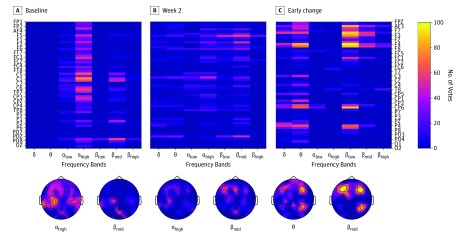
Number of Votes for Spectral Power Features The figure shows rankings for only 3 of 4 feature sources: baseline (A), week 2 (B), and early change (C). Combined feature source was the union of baseline and early change sources; therefore, computing the rankings for the combined features yielded results that were similar to the results for the baseline and early change sources. For that reason, feature rankings for combined feature source are not shown. The matrices in the top row display rankings for all the features; the topographic plots (left ear is left, nose is up) in the bottom row show distribution over the scalp of the rankings for the features corresponding to some chosen frequency bands.

Patterns that were less clearly structured were observed for spectral power features. As Figure 2 shows, the predictive features preferentially appeared in the high alpha and middle beta bands in the baseline and week 2 data and in the theta and low beta in the early change features. The asymmetry of multiscale entropy and power also provided a significant number of predictive features; however, these features were less clearly structured (eFigure 1 and eFigure 2 in the Supplement).

Current source density analysis revealed that high-alpha-band power in anterior cingulate cortex was the most prominent predictive feature shared by all the feature sources. In addition, high-alpha-band power in rostral anterior cingulate cortex appeared in baseline and week 2 data and high-beta-band at week 2 only (eFigure 3 in the Supplement). Microstate features did not exhibit any meaningful predictive capacity; the largest number of votes attained by any of the microstates’ features was 9.

### Classifier Construction and Performance Estimation

The prediction accuracy of SVMs for different feature sources and vote thresholds is summarized in Table 2. Increasing the number of features (by lowering the threshold) resulted in higher estimates of the model’s accuracy, with the early change feature source exhibiting an exception. The threshold of 60 votes provided a good trade-off between model complexity and performance; thus, we used this value for the rest of the analysis in this study. The eTable in the Supplement describes the classification performance for this threshold in more detail, including specificity and sensitivity values.

**Table 2.** Number of Features and Prediction Accuracy for Different Vote Thresholds and Feature Sources

Vote Threshold	Balanced Accuracy (No. of Features for a Particular Feature Source), % (No.)^a^			
	Baseline	Week 2	Early Change	Combined^b^
≥50	78.6 (54)	79.7 (75)	68.4 (149)	83.3 (185)
≥60	79.2 (34)	74.3 (50)	68.4 (106)	82.4 (127)
≥70	77.3 (17)	71.4 (28)	74.2 (73)	76.9 (82)
≥80	73.6 (9)	72.8 (14)	77.1 (50)	72.5 (52)
≥90	68.6 (4)	68.8 (5)	70.7 (20)	72.7 (21)

^a^ Balanced accuracy is defined as the mean of sensitivity and specificity.

^b^ Because the combined feature source was the union of baseline and early change feature sources, the feature count for the combined source was similar to the sum of the counts for baseline and early change sources. The count was similar rather than equal owing to the noise introduced into the feature ranking procedure by randomly choosing the 80% of the data to which the 2-tailed, unpaired *t* test was applied at each of the 100 iterations (see the Feature Ranking subsection of the Methods section).

The balanced accuracy of the classifier, as estimated by cross-validation, varied from 68.4% for early change feature source to 82.4% for combined feature source. Baseline-only data allowed prediction with an estimated balanced accuracy of 79.2% (sensitivity, 67.3%; specificity, 91.0%), whereas addition of week 2 data increased the accuracy to 82.4% (sensitivity, 79.2%; specificity, 85.5%). When computing sensitivity and specificity, we considered responder status to be a positive outcome and nonresponder to be a negative one.

### Assessment of Generalizability Across Sites

The results of the cross-site generalizability assessment are summarized in Table 3. The classification accuracy measured by the leave-one-site-out procedure was, in general, similar to that measured by 10-fold cross-validation on the complete data sample. However, the early change feature source again deviated from the general pattern by yielding inferior performance on the complete data sample.

**Table 3.** Cross-site Generalizability of the Treatment Outcome Prediction

Feature Source	Balanced Prediction Accuracy Testing on Data From a Single Site^a^^,^^b^								Balanced Prediction Accuracy^a^^,^^c^	
	UBC		TGH		QNS		CAM			
	Accuracy, %	Individuals, No.	Accuracy, %	Individuals, No.	Accuracy, %	Individuals, No.	Accuracy, %	Individuals, No.	Accuracy, %	Individuals, No.
Baseline	62.0	52	71.0	45	82.7	18	74.9	7	79.2	122
Week 2	74.5	51	74.7	40	79.2	18	72.0	6	74.3	115
Early change	69.9		85.0		77.4		78.6		68.4	
Combined	71.3		94.6		83.1		77.4		82.4	

Abbreviations: CAM, Centre for Addiction and Mental Health; QNS, Queens University; TGH, Toronto General Hospital; UBC, University of British Columbia.

^a^ Balanced prediction accuracy is defined as the mean of sensitivity and specificity.

^b^ Training was performed on data from the remaining sites.

^c^ Training and testing were performed on the data from all 4 sites using 10-fold cross-validation.

## Discussion

In this study, we demonstrated feasibility of predicting an outcome of escitalopram treatment using resting-state EEG. Of note, the prediction was that of the treatment outcome and not of the patient’s response to escitalopram. Without a properly randomized clinical trial, it is impossible to definitely attribute predicted differences in treatment outcomes to differences in individual patients’ responses to escitalopram in our particular experimental setup. However, more generally, there is a wide consensus^44,45,46^ regarding the assumption that variability in patients’ responses to escitalopram at least partially explains the variability of treatment outcomes. Together with this assumption, our results suggest that the predictive performance attained by our model is likely to at least partially involve prediction of the patient’s response to escitalopram. Of note, our study was not designed as an exhaustive optimization effort to achieve maximum possible classification accuracy. Such an effort would require a search over a larger space of candidate models (including models other than RBF kernel-based SVMs) and would demand larger data sets.

As hypothesized, combining baseline neural dynamics with early changes in neural dynamics (change after 2 weeks of treatment) resulted in the most accurate prediction. Results from leave-one-site-out cross-validation also demonstrated that the large-scale analysis of data pooled across multiple sites did not have a significant effect on classifier performance. This finding is important because it indicates the method’s robustness to variability stemming from differences in instrumentation and operation procedures across different clinical sites, making it more attractive for clinical translation.

A number of features identified through the feature selection process in this study have been previously reported to predict antidepressant response in patients with MDD. These features include parietal alpha,^19,20,47^ anterior cingulate cortex activity,^48,49,50^ and frontal theta.^18,21^ Our study also identified additional features that have not been previously reported, such as asymmetry in complexity of neural activity between hemispheres. This discovery of new EEG features that predict treatment outcome was a result of systematic evaluation of a large number of candidate measures using a relatively large data sample.

Machine learning studies using resting-state EEG measures for antidepressant response prediction are scarce. In the study by Khodayari-Rostamabad et al,^51^ resting-state EEG measures were used with a mixture of factor analysis classifier to predict antidepressant response. An accuracy of 87.9% was reported (sensitivity, 94.9%; specificity, 80.9%). Accuracy was also high (85%-92%) in the study by Rabinoff et al,^15^ which used spectral EEG features with classification and regression tree analysis. The study combined trials for 2 antidepressants (fluoxetine and venlafaxine) to predict response in 51 patients with unipolar depression. When interpreting the accuracy of the prediction models reported by different studies and especially comparing the accuracy across studies, a number of caveats should be considered. The most important one is that the reported accuracy numbers are usually estimated from data that are not strictly independent from the data used to fit the model parameters. Essentially this means that the estimates might be biased upward as a result of overfitting. Although a number of techniques are usually used to reduce this bias, such techniques do not necessarily eliminate it completely.^52,53^ The severity of the bias depends on many factors, such as sample size, number of free parameters in the model, and the bias-control techniques used. As a result, the bias is difficult to estimate and might vary greatly from study to study, making a direct comparison of reported accuracies misleading. Another important factor to consider when comparing results of different studies is the limitation on the maximal achievable accuracy that results from the noise in the target variable. The definition of response to treatment is typically grounded by a behavioral assessment summarized in a numerical variable (MADRS score in our study). However, this variable is inherently noisy; that is, the behavioral assessment of the same patient conducted twice yields somewhat different values.^54^ Consequently, it is impossible to predict such a variable (and, by extension, other variables derived from it, such as responder or nonresponder classification) with 100% accuracy. The upper bound on achievable accuracy depends on the amount of noise in the target variable, which might vary from study to study; the prediction accuracy reported by the study should be judged relative to that bound and not relative to the bound of 100%, which makes an unrealistic assumption of zero noise in the target labels.

To assess the practical relevance of the above argument, we computed an estimate of the target noise–induced bound for our sample (eMethods in the Supplement). The resulting estimate was 86.7%, indicating that the phenomenon was nonnegligible. Of importance, when computing that value, we made a number of arbitrary assumptions (such as modeling the MADRS noise with a normally distributed random variable); therefore, the result should be treated as a general estimate of the possible size of the effect rather than a measure of the effect in our particular study.

### Limitations

This study has limitations. Because this was an open-label study that lacked a control group, it was incapable of probing any causal relationships between the variables. Although we used a number of measures to control the overfitting, such as cross-validation and feature selection techniques, our performance estimate is not perfectly unbiased. Although our study used a larger data sample than most previous works, it was still limited, precluding model validation with a strictly independent data set. To obtain an unbiased estimate of our model’s predictive performance, future studies should validate it against an independent data set that was not used for model construction. This may be in part achieved through the next CAN-BIND validation study that aims to replicate the entire CAN-BIND-1 study in an independent cohort of patients. In addition, prediction models reported in this study have yet to be proven to be generalizable to other antidepressants. Model evaluation should be performed independently with several other types of treatment to evaluate the generalizability of our prediction models. This evaluation may also provide insight into whether the features identified in this study were specific to escitalopram treatment or whether they can be used with other pharmacologic agents. Performance may also be improved with the addition of clinical or behavioral variables, genetic measures, and other imaging-based measures (eg, functional magnetic resonance imaging and diffusion tensor imaging).

## Conclusions

This study provided a proof-of-concept pipeline for predicting changes in depression severity after the start of escitalopram treatment. Developed into a proper clinical application, such a pipeline may provide a valuable treatment planning tool. For large data sets that include several groups of patients, each receiving a different treatment option (pharmacologic and nonpharmacologic antidepressants), an approach similar to the one taken by this study may be useful in developing a model that can match each patient to the most effective treatment. The feasibility of such an approach will in part depend on the collection and sharing of large-scale, clinically reliable data sets, such as CAN-BIND. These investigations would contribute to the development of a clinical decision-making tool for data-driven, personalized optimization of antidepressant treatment selection for patients.

## Notes

Supplement. **eMethods.** Supplementary Methods **eFigure 1.** Rankings of MSE Asymmetry Features **eFigure 2.** Rankings of Electrode-Level Spectral Asymmetry Features **eFigure 3.** Rankings of Source-Level Spectral Features **eTable.** Characteristics of Classifiers Constructed From Features With ≥60 Votes **eReferences**
