# Supplementary material for: Use of Machine Learning for Predicting Escitalopram Treatment Outcome From Electroencephalography Recordings in Adult Patients With Depression
Source: JAMA Netw Open. 2020 Jan 3;3(1):e1918377. doi: 10.1001/jamanetworkopen.2019.18377 (PMC6991244; doi:10.1001/jamanetworkopen.2019.18377)
Supplement: Supplement. — eMethods. Supplementary Methods eFigure 1. Rankings of MSE Asymmetry Features eFigure 2. Rankings of Electrode-Level Spectral Asymmetry Features eFigure 3. Rankings of Source-Level Spectral Features eTable. Characteristics of Classifiers Constructed From Features With ≥60 Votes eReferences [file jamanetwopen-3-e1918377-s001.pdf]

Supplementary Online Content

Zhdanov A, Atluri S, Wong W, et al. Use of machine learning for predicting escitalopram treatment outcome from electroencephalography recordings in adult patients with depression. *JAMA Netw Open*. 2020;3(1):e1918377. doi:10.1001/jamanetworkopen.2019.18377

eMethods. Supplementary Methods

eFigure 1. Rankings of MSE Asymmetry Features

eFigure 2. Rankings of Electrode-Level Spectral Asymmetry Features

eFigure 3. Rankings of Source-Level Spectral Features

eTable. Characteristics of Classifiers Constructed From Features With ≥60 Votes

eReferences

This supplementary material has been provided by the authors to give readers additional information about their work.

© 2020 Zhdanov A et al. *JAMA Network Open.*

eMethods. Supplementary Methods

Participants

In the first Canadian Biomarker Integration Network in Depression (CAN-BIND-1) study[1], participants (n=211) aged 18-60 years who met the DSM-IV requirements for major depressive disorder and a current major depressive episode were recruited for the study. The study was conducted across six sites. Out of 211 patients, 180 completed 8 weeks of standardized escitalopram treatment (10-20mg). At week 8, responder or non-responder status was determined based on decrease in MADRS score from baseline (≥50% for responders and <50% for non-responders, see *Kennedy et al.*[1] for further details) and patients continued with treatment for another 8 weeks (escitalopram or escitalopram + aripiprazole). At four out of the six participating sites, EEG assessments were also conducted over the course of the study. These sites included: University of British Columbia (UBC), Toronto General Hospital (TGH), Queens University (QNS) and the Centre for Addiction and Mental Health (CAM). EEG measurements were performed at baseline (within 3 days before the start of the treatment trial), and then after 2 and 8 weeks of treatment. During each measurement a single *dataset*, containing approximately eight minutes of resting-state, eyes-closed data, was recorded per patient. This study used:

1. 122 datasets recorded at baseline for 122 out of 180 patients, and

2. 115 datasets recorded at week 2 for 115 out of the 122 patients that completed the baseline recording

We did not use EEG data recorded at week 8 because of the limited clinical value of predicting treatment response that late into the treatment.

Demographic and clinical data of the participants is summarized in Tables 1-3 below. Response and non-response over the entire trial are illustrated in Figures 1-2 below. Detailed description of the clinical data, research protocol and data acquisition at each site was provided in previously published work[1–3].

Table 1. Demographic and Clinical Data for All the Subjects That Completed 8 Weeks of Treatment

Clinical/ demographic values	Clinical site[1]						All	All responders (Week 8)	All non-responders (Week 8)
	UBC	TGH	QNS	CAM	MCU	UCA			
N	54	47	18	7	28	26	180	85	95
Age, mean (SD)	35.6 (11.9)	35.7 (12.8)	42.7 (14.0)	30.4 (12.1)	34.0 (12.4)	32.5 (11.3)	35.4 (12.7)	35.0 (12.1)	35.8 (13.1)
Sex (M/F)	19/35	19/28	9/9	0/7	8/20	13/13	68/ 112	30/55	38/57
MADRS, baseline, mean (SD)	28.3 (5.8)	32.2 (5.5)	30.0 (4.6)	28.0 (4.8)	29.2 (5.2)	30.9 (4.3)	30.0 (5.5)	29.5 (5.5)	30.5 (5.4)
MADRS, week 2, mean (SD)	21.9 (7.2)	25.1 (10.6)	23.1 (5.2)	21.1 (3.7)	18.7 (7.4)	23.9 (7.5)	22.6 (8.3)	19.6 (8.2)	25.4 (7.5)
MADRS, week 8, mean (SD)	14.2 (9.2)	19.2 (11.9)	18.1 (9.9)	15.7 (5.6)	14.6 (8.8)	15.9 (9.1)	16.3 (10.1)	8.0 (5.0)	23.7 (7.3)
Decrease in MADRS (baseline to week 8), mean (SD)	14.1 (9.1)	13.0 (11.7)	11.9 (9.9)	12.3 (4.1)	14.6 (8.7)	15.1 (8.8)	13.7 (9.8)	21.5 (6.2)	6.8 (6.6)
Decrease in MADRS (baseline to week 8), relative to baseline, mean (SD), %	49.9 (32.6)	40.6 (32.7)	39.2 (34.9)	44.5 (15.7)	50.0 (29.0)	49.1 (28.9)	46.1 (31.7)	73.1 (15.6)	21.9 (21.0)
Responders / Non-responders (after 8 weeks)	26/28	21/26	7/11	3/4	15/13	13/13	85/95	-	-

[1] The table uses the following abbreviations for site names: UBC—University of British Columbia, TGH—Toronto General Hospital, QNS—Queens University, CAM—Centre for Addiction and Mental Health, MCU—McMaster University, UCA—University of Calgary. Note that sites MCU and UCA did not conduct EEG recordings.

© 2020 Zhdanov A et al. *JAMA Network Open.*

Table 2. Demographic and clinical data for subjects in the baseline visit

Clinical/ demographic values	EEG recording site[2]				All	All responders (Week 8)	All non-responders (Week 8)
	UBC	TGH	QNS	CAM			
N	52	45	18	7	122	55	67
Age, mean (SD)	35.4 (11.5)	35.7 (12.6)	42.7 (14.0)	30.4 (12.1)	36.3 (12.7)	36.0 (12.7)	36.6 (12.6)
Sex (M/F)	19/33	18/27	9/9	0/7	46/76	19/36	27/40
MADRS, baseline, mean (SD)	28.5 (5.8)	32.3 (5.6)	30.0 (4.6)	28.0 (4.8)	30.1 (5.8)	29.5 (5.8)	30.5 (5.8)
MADRS, week 2, mean (SD)	21.9 (7.3)	25.1 (10.1)	23.1 (5.2)	21.1 (3.7)	23.2 (8.5)	20.1 (8.4)	25.8 (7.8)
MADRS, week 8, mean (SD)	14.6 (9.2)	19.1 (12.0)	18.1 (9.9)	15.7 (5.6)	16.8 (10.5)	7.9 (5.0)	24.2 (7.7)
Decrease in MADRS (baseline to week 8), mean (SD)	13.9 (9.2)	13.2 (11.8)	11.9 (9.9)	12.3 (4.1)	13.2 (10.2)	21.7 (6.7)	6.3 (6.8)
Decrease in MADRS (baseline to week 8), relative to baseline, mean (SD), %	48.7 (32.7)	41.1 (3.8)	39.2 (34.9)	44.5 (15.7)	44.3 (32.6)	73.3 (16.0)	20.4 (21.6)
Responders / Non-responders (after 8 weeks)	24/28	21/24	7/11	3/4	55/67	-	-

[2] The table uses the following abbreviations for site names: UBC—University of British Columbia, TGH—Toronto General Hospital, QNS—Queens University, CAM—Centre for Addiction and Mental Health

© 2020 Zhdanov A et al. *JAMA Network Open.*

Table 3. Demographic and clinical data for subjects in the week 2 visit

Clinical/ demographic values	EEG recording site[3]				All	All responders (Week 8)	All non-responders (Week 8)
	UBC	TGH	QNS	CAM			
N	51	40	18	6	115	53	62
Age, mean (SD)	35.8 (11.4)	35.5 (12.3)	42.7 (14.0)	26.0 (5.7)	36.2 (12.4)	35.3 (12.5)	37.0 (12.3)
Sex (M/F)	19/32	15/25	9/9	0/6	43/72	19/34	24/38
MADRS, baseline, mean (SD)	28.5 (5.9)	32.2 (5.8)	30.0 (4.6)	28.8 (4.7)	30.0 (5.9)	29.5 (5.7)	30.5 (5.9)
MADRS, week 2, mean (SD)	21.9 (7.4)	25.0 (11.2)	23.1 (5.2)	21.8 (3.5)	23.2 (8.6)	20.1 (8.5)	25.8 (7.8)
MADRS, week 8, mean (SD)	14.2 (8.8)	18.4 (12.1)	18.1 (9.9)	17.0 (5.0)	16.4 (10.3)	7.8 (5.0)	23.8 (7.5)
Decrease in MADRS (baseline to week 8), mean (SD)	14.3 (8.8)	13.9 (11.8)	11.9 (9.9)	11.8 (4.3)	13.6 (10.0)	21.8 (6.8)	6.7 (6.5)
Decrease in MADRS (baseline to week 8), relative to baseline, mean (SD), %	50.3 (31.1)	43.2 (32.6)	39.2 (34.9)	41.1 (14.3)	45.6 (31.9)	73.6 (16.2)	21.6 (20.5)
Responders / Non-responders (after 8 weeks)	24/27	20/20	7/11	2/4	53/62	-	-

[3] The table uses the following abbreviations for site names: UBC—University of British Columbia, TGH—Toronto General Hospital, QNS—Queens University, CAM—Centre for Addiction and Mental Health

© 2020 Zhdanov A et al. *JAMA Network Open.*

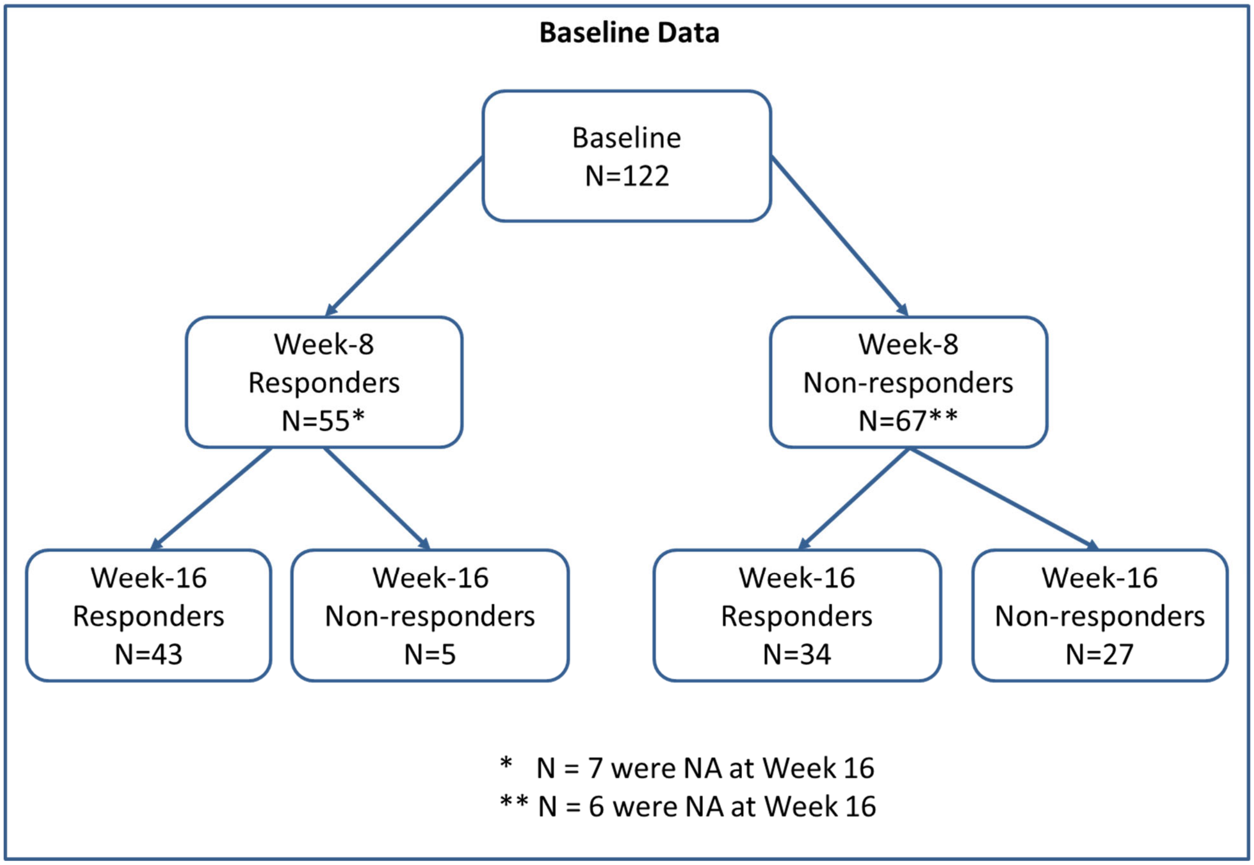

Figure 1. Response over 16 weeks of the CAN-BIND-1 trial for the 122 subjects with baseline data included in this study. At week 8, response was defined as ≥ 50% in MADRS score from baseline to week 8. At week 16, response was defined as ≥ 50% in MADRS score from baseline to week 16.

© 2020 Zhdanov A et al. *JAMA Network Open.*

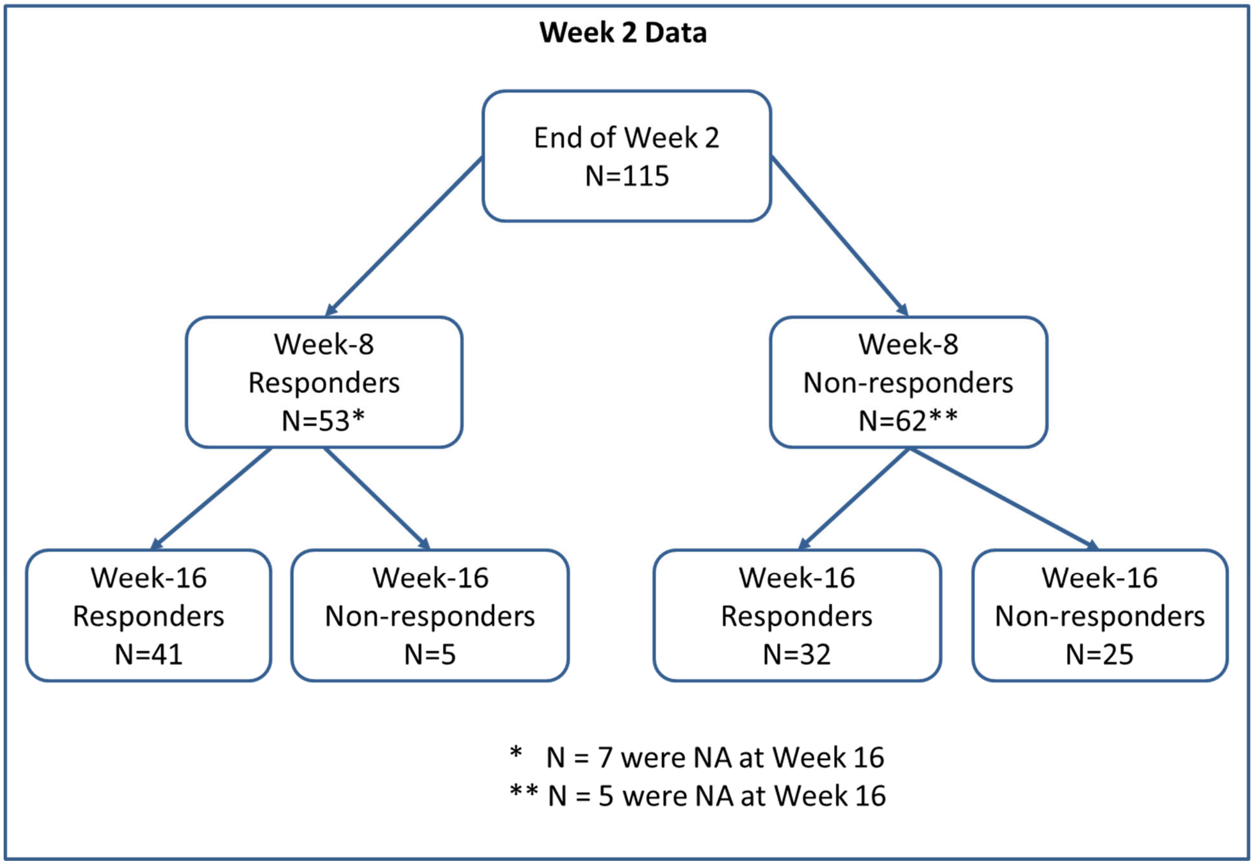

Figure 2. Response over 16 weeks of the CAN-BIND-1 trial for the 115 subjects with baseline and week 2 data included in this study. At week 8, response was defined as ≥ 50% in MADRS score from baseline to week 8. At week 16, response was defined as ≥ 50% in MADRS score from baseline to week 16.

Inter-site Data Harmonization and Pre-processing

All EEG datasets were standardized to the following parameters: 58 electrodes common to the equipment across all sites, 0.05-100Hz bandpass filter, average reference, 512 Hz sampling rate. All the filtering was done using 2nd order Butterworth filters applied to the data twice—in forward and reverse direction, to eliminate any phase distortion. The standardization was performed with the EEGLAB toolbox (v12.0.2.6b)[4]. Complete descriptions of the EEG standardization procedure across sites employed by the current study can be found in *Farzan et al.*[5].

Data pre-processing was conducted with EEGERP toolbox[5]. Channels contaminated by large sporadic artifact were identified by human analyst and deleted. EEG data were bandpass-filtered between 1 and 80 Hz, and notch-filtered at 60 Hz. Independent component analysis was used to remove eye movements and blinks, electromyography, and electrode discontinuity artifacts. The deleted EEG channels were interpolated using spherical spline interpolation[6].

Feature Computation

Once the data was harmonized and pre-processed, we proceeded to feature computation. For each feature, we segmented the data into continuous intervals of equal length, computed the feature for each interval independently, and averaged the results to summarize the feature in a single value per patient. We used 2-second-long intervals for all the features except for MSE, for which interval duration of 20 seconds was chosen based on previous studies[7,8].

The four classes of features used in this study are defined as follows:

1. Electrode-level spectral features

   We computed electrode-level spectral features by using EEGLAB function *spectopo* to obtain the power spectrum for each electrode. The log-transformed absolute power was obtained for each channel for each of

© 2020 Zhdanov A et al. *JAMA Network Open.*

the frequency bands defined above. Asymmetry between left and right hemispheres was also considered. To that end, all the electrodes located off the midline (50 electrodes in our montage) were split into 25 pairs symmetrical with the respect to the midline, e.g. FP1/FP2, AF3/AF4, etc. For each pair, the absolute power at the left electrode was divided by the absolute power at the right electrode, resulting in 25 features for each frequency band, describing the lateralization of EEG power.

2. Source-level spectral features

   Current source density analysis was performed using the eLORETA algorithm as implemented by the LORETA-KEY software[9]. The transformation matrix (58 channels to 6239 voxels) was derived by co-registering electrode co-ordinates (10-10 international system) to the MNI152 MRI head template[10] using a relative regularization parameter of 1. The solution space of eLORETA was restricted to cortical and some hippocampal and amygdala grey matter. The MNI152 template brain volume was divided into 6239 cortical gray matter voxels at 5-mm^3 resolution[10]. From scalp-recorded electrical potential distribution, LORETA computes the three-dimensional intracerebral distributions of current density for each of the frequency bands specified above. The following regions were selected based on the previous literature: the anterior cingulate cortex (ACC), rostral ACC (rACC), and the medial orbitofrontal cortex (mOFC)[11,12].

3. Multiscale-entropy-based features

   Multiscale entropy analysis was performed using previously described methods[13,14]. Using the sample entropy equation, multiscale entropy was examined across all the 58 electrodes with the coarse-graining process for 70 scales. Sample entropy quantifies the variability of time series by estimating the predictability of amplitude patterns across a time series. In our analysis, two consecutive data points were used for data matching (m=2) and data points were considered to match if their absolute amplitude difference was less than 15% (i.e., r=0.15) of the standard deviation of the time series (similar to the previous literature[13,14]).

   We have also computed asymmetry between left and right hemispheres, in a fashion similar to computing the asymmetry for the power at specific frequency bands (see above): multiscale entropy in the left hemisphere was divided by the multiscale entropy in the right hemisphere for the 25 channel pairs: FP1/FP2, AF3/AF4, …, and O1/O2.

4. Microstate-based features

   Microstate analysis followed the standard procedure outlined elsewhere[15,16] and was implemented using CARTOOL[17]. Prior to the application of microstate analysis, pre-processed EEG data were bandpass-filtered from 1 to 30 Hz. The topographical maps at the local maxima peaks of the global field power curve were clustered to derive the four prototypical microstate classes[18]. In this study, the topographical atomize–agglomerate hierarchical clustering algorithm[19] was applied to cluster samples from each EEG dataset into four states (microstate maps). Finally, topographical maps at each local maxima point of the global field power curve were assigned to the microstate class of highest correlation using spatial Pearson's product-moment correlation coefficient[20]. Three features were calculated for each of the four microstate classes: (i) average duration, (ii) frequency, and (iii) coverage. Average duration refers to the average amount of time a microstate class remains stable when it appears, in milliseconds; frequency refers to the occurrence of each microstate class per second; and coverage is the percent of recording covered by each microstate class.

Baseline and Week 2 sources comprise 6424 features each: 581 electrode-level spectral features (7 bands x 58 channels + 7 bands x 25 channel pairs), 5810 multiscale-entropy-based features (70 scales x 58 channels + 70 scales x 25 channel pairs), 12 microstate-based features (4 maps x 3 features) and 21 source-level spectral features (7 bands x 3 regions).

Features for the Early Change source are calculated as (post-pre)/pre for multiscale-entropy-based, microstate-based and source-level spectral features and (post-pre) for electrode-level spectral features.

The total number of features for the Early Change source was 6424. For the Combined source, the total number of features was (6424+6424) 12,848.

Feature Ranking

We used a *t*-test-based filtering method to rank the features according to their predictive power. The method repeats the steps described below for 100 iterations:

1. Generate a training set by randomly selecting 80% of responders and 80% of non-responders from the whole data sample.
2. For each feature, test whether the feature's value is significantly different between responders and non-responders in the training set only, using *t*-test. If the test passes the significance threshold at $\alpha<0.05$, the iteration counts as one vote for the feature, otherwise—as 0 votes.

The total number of votes for each feature over the 100 iteration therefore varies between 0 and 100, with more votes indicating the features that are more robustly predictive of the treatment outcome.

Classifier Construction and Performance Estimation

SVM is a binary classifier that utilizes a hyperplane (linear SVM) or hypersurface (non-linear SVM) that best separates the input feature space into the two pre-defined groups (in our case, responders and non-responders)[21]. An RBF kernel uses nonlinear mapping to transform data into a higher dimensional space and determine an optimal hypersurface for classification[22]. The optimal hypersurface separates two groups with the largest margin (i.e., distance between the hypersurface and the closest data points). In this study we use LIBSVM toolbox's[23] implementation of RBF SVMs.

RBF version of SVM constitutes a family of classification algorithms parameterized by two hyperparameters, denoted here as C (also known as penalty) and γ. Each particular combination of values of C and γ essentially yields a different classifier with different learning behavior. To find a combination of (C, γ) that yields an optimal classifier for our data, we use a grid-search method[23] over a restricted range of C and γ values:

1. We restrict the range of potential values to $C \in \{2^{-3}, 2^{-1}, 2^{1}, 2^{3}\}$, and $\gamma \in \{2^{-12}, 2^{-10}, 2^{-8}, 2^{-6}\}$
2. Using 10-fold cross-validation, we estimate balanced accuracy of predicting the treatment outcome for each of the resulting 16 parameter combinations.
3. We select the combination yielding the best estimate.

We use the above procedure to estimate the balanced accuracy of SVM prediction of treatment outcome for the four feature sources (Baseline, Week 2, Early Change and Combined) and several different vote thresholds $T \in \{50, 60, 70, 80, 90\}$.

Assessment of Bounds on Achievable Classification Accuracy

Prediction accuracy of any classifier is limited by the intrinsic noise of the target variable. In our case, the target variable–treatment response–is derived from MADRS scores, which are known to contain significant noise. To assess the impact of the imperfect reliability of MADRS scoring procedure on our study, we performed the simulation described below.

Consider a hypothetical perfect classifier that achieves 100% classification accuracy on all the data in our disposal. This accuracy is achieved w.r.t. the binary target variable (responder/non-responder) that was computed from MADRS scores obtained from the patients. However, imperfect reliability of the scoring procedure means that if we were to repeat the scoring for the same patients and the same levels of depression severity, we would obtain somewhat different results. This, in turn, would lead to a somewhat different assignment of responder/non-responder labels to the patients. If we were to test our perfect classifier on this alternative target labels, we would observe a classification accuracy of less than 100%. This degraded classification accuracy reflects the inherent limitation on possible performance posed

by the noise in the target variable rather than shortcoming of any particular classifier design. It also provides an upper bound on the performance achievable by the best possible model trained on an infinite amount of data.

To carry out the above simulation in practice, we first need a procedure for realistic modeling of MADRS scoring noise. We used a (somewhat optimistic) estimate of the MADRS scoring reliability reported by *Williams and Kobak*[24]. In this study the researchers conducted two identical scoring sessions on each of the study participants and compared the scores. The similarity between two datasets, as measured by Intra-Class Correlation (ICC), was 0.93.

We have generated 10,000 surrogate instances of the MADRS scores by adding a random Gaussian noise to the original scores (for both week 0 and week 8). The noise had zero mean and the variance was selected in such a way that the average ICC between the original and the surrogate scores was about 0.93. For each surrogate instance, we computed the classification error of the "perfect" classifier – the one that attains 100% accuracy on the original data. Introducing the noise into MADRS data on average degraded the maximal achievable classification accuracy from 100% to 86.7% (with standard deviation of 2.8%).

eFigure 1

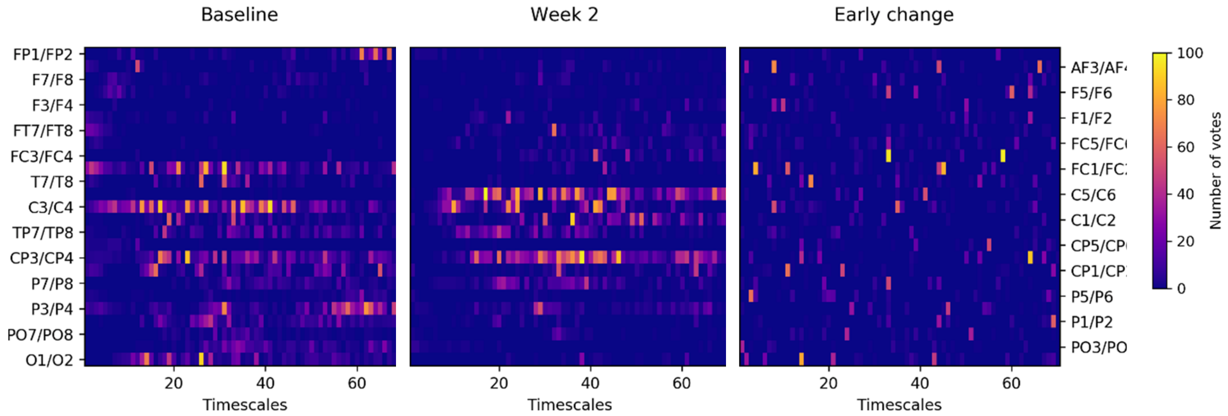

eFigure 1. Rankings of MSE Asymmetry Features.
The figure only shows rankings for 3 out of 4 feature sources (Baseline, Week 2, and Early Change). Combined feature source is the union of Baseline and Early Change sources; therefore computing the rankings for the Combined features yields results that are very similar to the results for the Baseline and Early Change sources. For that reason, feature rankings for Combined feature source are not shown.
The horizontal axis represents MSE scales; the vertical enumerates pairs of symmetrically located electrodes. The color of each square indicates the number of votes accrued by the corresponding feature.

© 2020 Zhdanov A et al. *JAMA Network Open.*

eFigure 2

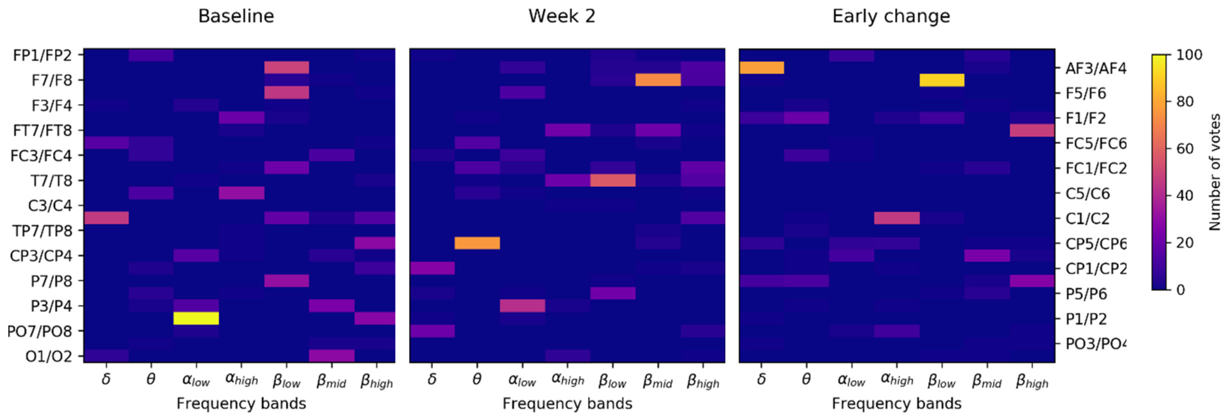

eFigure 2. Rankings of Electrode-Level Spectral Asymmetry Features
The figure only shows rankings for 3 out of 4 feature sources (Baseline, Week 2, and Early Change). Combined feature source is the union of Baseline and Early Change sources; therefore computing the rankings for the Combined features yields results that are very similar to the results for the Baseline and Early Change sources. For that reason, feature rankings for Combined feature source are not shown.
The horizontal axis represents frequency bands; the vertical enumerates pairs of symmetrically located electrodes. The color of each square indicates the number of votes accrued by the corresponding feature.

eFigure 3

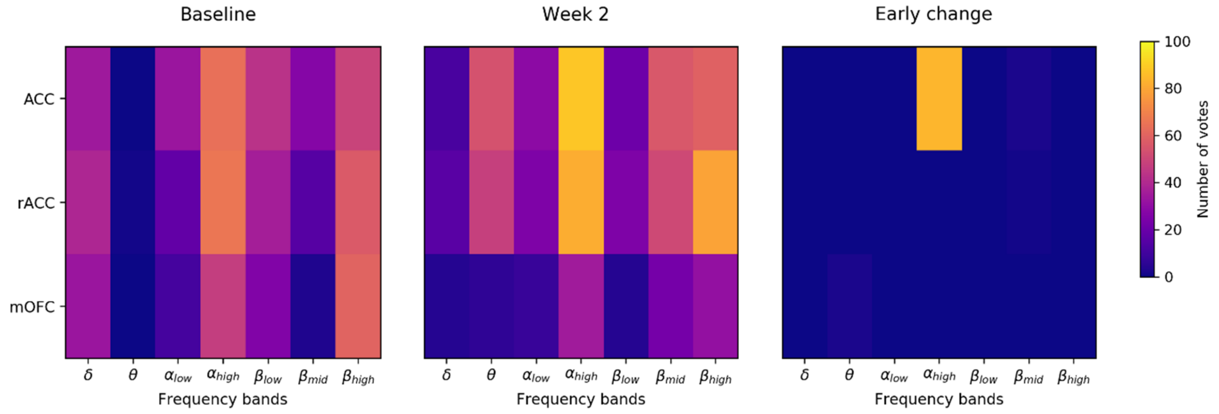

eFigure 3. Rankings of Source-Level Spectral Features
The figure only shows rankings for 3 out of 4 feature sources (Baseline, Week 2, and Early Change). Combined feature source is the union of Baseline and Early Change sources; therefore computing the rankings for the Combined features yields results that are very similar to the results for the Baseline and Early Change sources. For that reason, feature rankings for Combined feature source are not shown.
The horizontal axis represents frequency bands; the vertical enumerates brain regions (ACC - anterior cingulate cortex, rACC - rostral ACC, mOFC - medial orbitofrontal cortex). The color of each square indicates the number of votes accrued by the corresponding feature.

© 2020 Zhdanov A et al. *JAMA Network Open.*

eTable. Characteristics of Classifiers Constructed From Features With ≥60 Votes

	Feature source			
	Baseline	Week 2	Early Change	Combined
**Number of features**	34	50	106	127[4]
**Sensitivity, %**	67.3	67.9	52.8	79.2
**Specificity, %**	91.0	80.6	83.9	85.5
**Balanced accuracy, %**	79.2	74.3	68.4	82.4

eReferences

1. Kennedy SH, Lam RW, Rotzinger S, et al. Symptomatic and Functional Outcomes and Early Prediction of Response to Escitalopram Monotherapy and Sequential Adjunctive Aripiprazole Therapy in Patients With Major Depressive Disorder. *The Journal of Clinical Psychiatry*. 2019;80(2). doi:10.4088/JCP.18m12202

2. Lam RW, Milev R, Rotzinger S, et al. Discovering biomarkers for antidepressant response: protocol from the Canadian biomarker integration network in depression (CAN-BIND) and clinical characteristics of the first patient cohort. *BMC Psychiatry*. 2016;16:105. doi:10.1186/s12888-016-0785-x

3. Baskaran A, Farzan F, Milev R, et al. The comparative effectiveness of electroencephalographic indices in predicting response to escitalopram therapy in depression: A pilot study. *Journal of Affective Disorders*. 2018;227:542-549. doi:10.1016/j.jad.2017.10.028

4. Delorme A, Makeig S. EEGLAB: an open source toolbox for analysis of single-trial EEG dynamics including independent component analysis. *Journal of Neuroscience Methods*. 2004;134(1):9-21. doi:10.1016/j.jneumeth.2003.10.009

5. Farzan F, Atluri S, Frehlich M, et al. Standardization of electroencephalography for multi-site, multi-platform and multi-investigator studies: insights from the canadian biomarker integration network in depression. *Scientific reports*. 2017;7(1):7473. doi:10.1038/s41598-017-07613-x

6. Perrin F, Pernier J, Bertrand O, Echallier JF. Spherical splines for scalp potential and current density mapping. *Electroencephalography and Clinical Neurophysiology*. 1989;72(2):184-187. doi:10.1016/0013-4694(89)90180-6

7. Takahashi T, Cho RY, Murata T, et al. Age-related variation in EEG complexity to photic stimulation: a multiscale entropy analysis. *Clin Neurophysiol*. 2009;120(3):476-483. doi:10.1016/j.clinph.2008.12.043

8. Ueno K, Takahashi T, Takahashi K, Mizukami K, Tanaka Y, Wada Y. Neurophysiological basis of creativity in healthy elderly people: a multiscale entropy approach. *Clin Neurophysiol*. 2015;126(3):524-531. doi:10.1016/j.clinph.2014.06.032

[4] Since the Combined feature source is the union of Baseline and Early Change feature sources, the feature count for the Combined source is similar to the sum of the counts for Baseline and Early Change sources. The reason for it being similar rather than strictly equal is the noise introduced into the feature ranking procedure by randomly choosing the 80% of the data to which the t-test is applied at each of the 100 iterations (see Feature ranking in the Methods section of the manuscript).

© 2020 Zhdanov A et al. *JAMA Network Open.*

9. Pascual-Marqui RD, Lehmann D, Koenig T, et al. Low resolution brain electromagnetic tomography (LORETA) functional imaging in acute, neuroleptic-naive, first-episode, productive schizophrenia. *Psychiatry Research: Neuroimaging*. 1999;90(3):169-179. doi:10.1016/S0925-4927(99)00013-X

10. Pascual-Marqui RD. Standardized low-resolution brain electromagnetic tomography (sLORETA): technical details. *Methods Find Exp Clin Pharmacol*. 2002;24 Suppl D:5-12.

11. Korb AS, Hunter AM, Cook IA, Leuchter AF. Rostral anterior cingulate cortex theta current density and response to antidepressants and placebo in major depression. *Clinical Neurophysiology*. 2009;120(7):1313–1319. doi:10.1016/j.clinph.2009.05.008

12. Pizzagalli D, Pascual-Marqui RD, Nitschke JB, et al. Anterior Cingulate Activity as a Predictor of Degree of Treatment Response in Major Depression: Evidence From Brain Electrical Tomography Analysis. *American Journal of Psychiatry*. 2001;158(3):405–415. doi:10.1176/appi.ajp.158.3.405

13. Costa M, Goldberger AL, Peng C-K. Multiscale entropy analysis of biological signals. *Phys Rev E Stat Nonlin Soft Matter Phys*. 2005;71(2 Pt 1):021906. doi:10.1103/PhysRevE.71.021906

14. Farzan F, Atluri S, Mei Y, et al. Brain temporal complexity in explaining the therapeutic and cognitive effects of seizure therapy. *Brain : a journal of neurology*. 2017;140(4):1011–1025. doi:10.1093/brain/awx030

15. Pascual-Marqui RD, Michel CM, Lehmann D. Segmentation of brain electrical activity into microstates: model estimation and validation. *IEEE Trans Biomed Eng*. 1995;42(7):658-665. doi:10.1109/10.391164

16. Lehmann D, Ozaki H, Pal I. EEG alpha map series: brain micro-states by space-oriented adaptive segmentation. *Electroencephalogr Clin Neurophysiol*. 1987;67(3):271-288.

17. Brunet D, Murray MM, Michel CM. Spatiotemporal Analysis of Multichannel EEG: CARTOOL. Computational Intelligence and Neuroscience. doi:10.1155/2011/813870

18. Koenig T, Prichep L, Lehmann D, et al. Millisecond by Millisecond, Year by Year: Normative EEG Microstates and Developmental Stages. *NeuroImage*. 2002;16(1):41-48. doi:10.1006/nimg.2002.1070

19. Tibshirani R, Walther G. Cluster Validation by Prediction Strength. *Journal of Computational and Graphical Statistics*. 2005;14(3):511-528. doi:10.1198/106186005X59243

20. Brandeis D, Naylor H, Halliday R, Callaway E, Yano L. Scopolamine effects on visual information processing, attention, and event-related potential map latencies. *Psychophysiology*. 1992;29(3):315-336.

21. Hearst MA, Dumais ST, Osuna E, Platt J, Scholkopf B. Support vector machines. *IEEE Intelligent Systems and their Applications*. 1998;13(4):18-28. doi:10.1109/5254.708428

22. Hsu C-W, Chang C-C, Lin C-J. A Practical Guide to Support Vector Classification. 2003;101:1396-1400.

23. Chang C-C, Lin C-J. LIBSVM: A Library for Support Vector Machines. *ACM Trans Intell Syst Technol*. 2011;2(3):27:1–27:27. doi:10.1145/1961189.1961199

24. Williams JBW, Kobak KA. Development and reliability of a structured interview guide for the Montgomery Asberg Depression Rating Scale (SIGMA). *The British journal of psychiatry : the journal of mental science*. 2008;192(1):52–58. doi:10.1192/bjp.bp.106.032532
